# Reconstitution of disrupted photoreceptor layer in uveitis associated with Behçet’s disease by infliximab treatment

**DOI:** 10.1186/s12886-015-0162-4

**Published:** 2015-12-12

**Authors:** Masaru Takeuchi, Kozo Harimoto, Manzo Taguchi, Yutaka Sakurai

**Affiliations:** Department of Ophthalmology, National Defense Medical College, 3-2 Namiki, Tokorozawa City, Saitama 359-8513 Japan

**Keywords:** Behçet’s disease, Choroid, Optical coherence tomography, Uveitis, Infliximab

## Abstract

**Background:**

Behçet’s disease (BD)-associated uveitis causes retinal damage leading to severe visual disturbance. The early morphological changes in the retina are revealed by disappearance or disruption of the external limiting membrane (ELM), inner segment ellipsoid zone (EZ) and cone interdigitation zone (CIZ) in the outer retina shown on spectral domain-optical coherence tomography (SD-OCT). However, it is unknown whether these changes in the retina are reversible in BD-associated uveitis.

**Case presentation:**

A 38-year-old man was referred to our hospital with 5 years history of panuveitis in both eyes. Recurrent oral ulcer, folliculitis, and genital ulcer were noted as systemic complications. Moderate cell infiltration into the anterior chamber, and diffuse vitritis were observed in both eyes, and best corrective visual acuity (BCVA) was 20/60 in the right and 20/200 in the left eye. Fluorescein angiography (FA) showed severe dye leakage from extensive retinal vessels in both eyes. Spectral domain-optical coherence tomography (SD-OCT) revealed retinal cysts and disruption of the external limiting membrane (ELM), inner segment ellipsoid zone (EZ) and cone interdigitation zone (CIZ) in the macular region of both eyes. BD was diagnosed based on the ocular features and systemic lesions, and infliximab therapy was initiated for the severe visual disturbance. After treatment with infliximab, foveal excavation was first recovered with disappearance of retinal cysts, and then ELM and EZ were gradually reconstituted on SD-OCT. Finally, CIZ became distinguishable after 24 months of infliximab therapy. BCVA was recovered to 20/25 in both eyes, and ocular inflammatory attack did not recur after the initiation of infliximab therapy.

**Conclusion:**

Disruption of ELM, EZ, and CIZ shown on SD-OCT in BD-associated uveitis could be reconstituted by continuous infliximab treatment, which leaded to the improvement of visual acuity.

## Background

Behçet’s disease (BD)-associated uveitis is characterized by iridocyclitis with hypopyon and progressive retinal vasculitis. Ocular inflammatory attacks, occur more frequent for 4 years after the onset of uveitis, cause retinal damage leading to severe visual disturbance [1]. The early morphological changes in the retina are revealed by disappearance or disruption of the external limiting membrane (ELM), inner segment ellipsoid zone (EZ) and cone interdigitation zone (CIZ) in the outer retina shown on spectral domain-optical coherence tomography (SD-OCT). However, it is unknown whether these changes in the retina are reversible in BD-associated uveitis.

## Case presentation

**Fig. 1:**
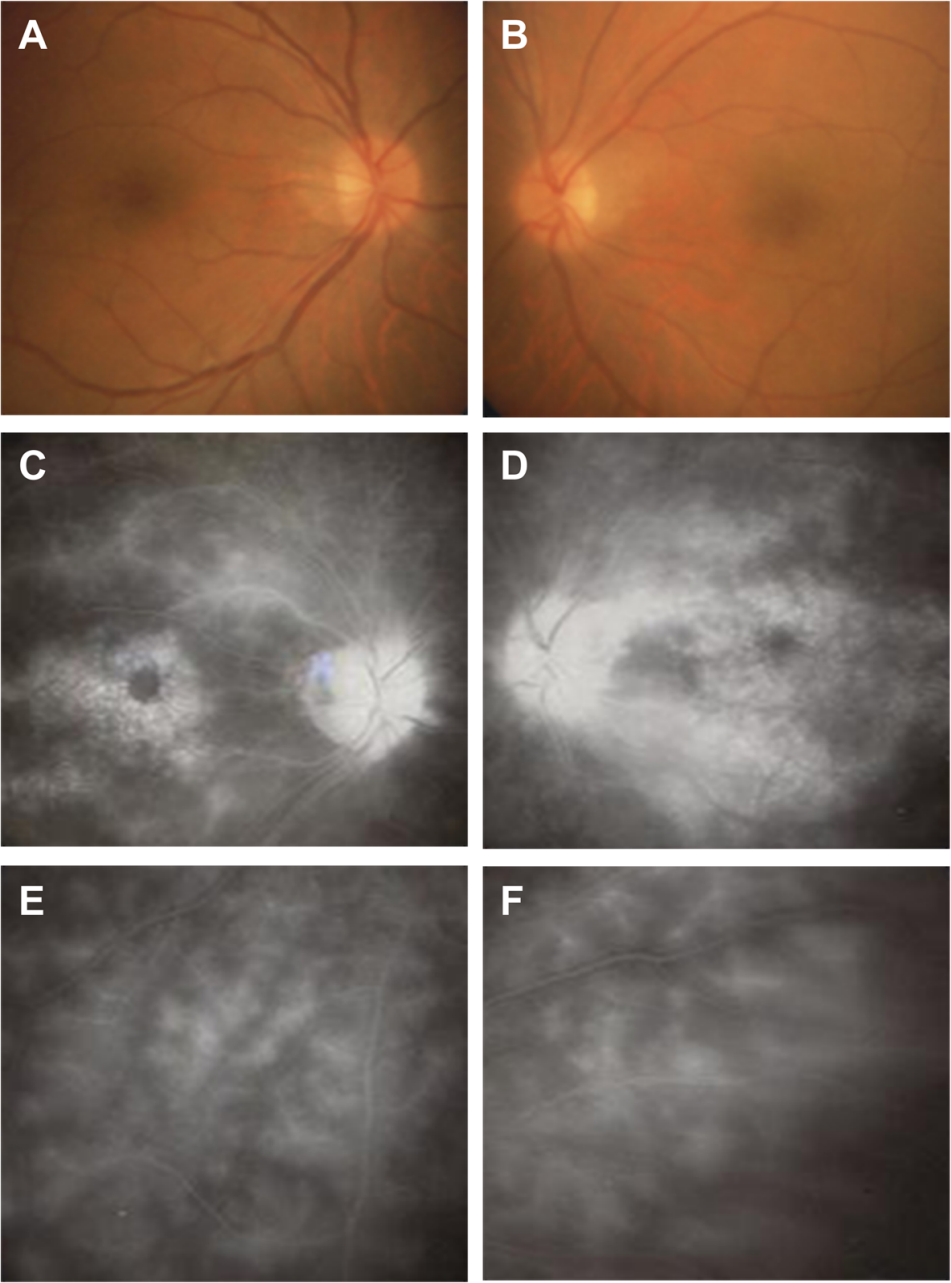
Color Fundus and Fluorescein Angiography photographs. Color fundus photographs show no definite retinal lesion in the right (**a**) and left (**b**) eyes. Late-phase fluorescein angiography reveals intensive dye leakage from vessels at the optic disc and macula in the right (**c**) and left (**d**) eyes, and in peripheral retina in the right (**e**) and left (**f**) eyes

**Fig. 2:**
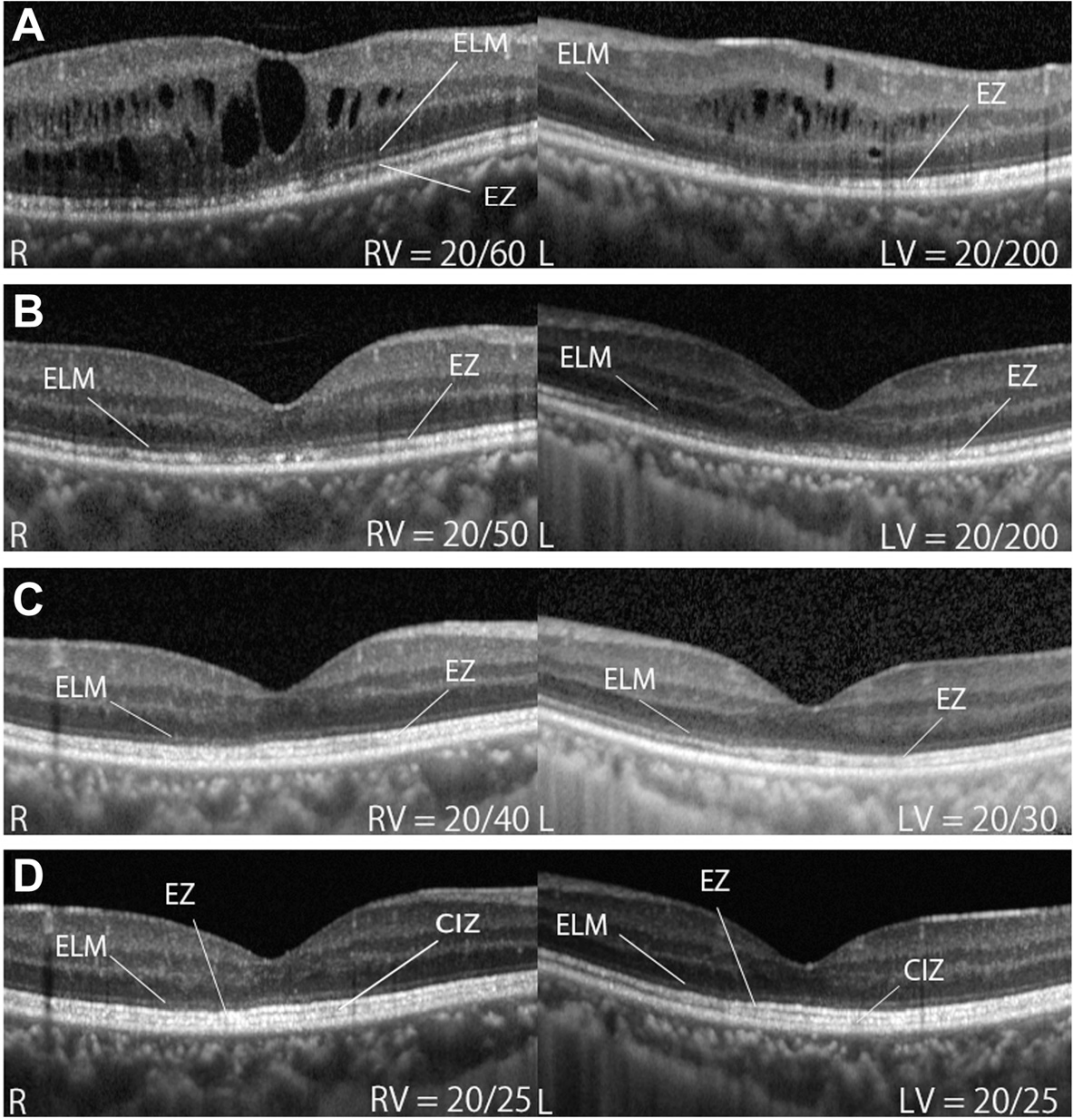
Spectral domain-optical coherence tomography (SD-OCT) images at the macula before and after infliximab therapy. The panels show SD-OCT images at the macula in the right (R) and left (L) eyes before infliximab therapy (**a**), and after 3 months (**b**), 12 months (**c**) and 24 months (**d**) of therapy. RV: Best corrected visual acuity in the right eye, LV: best corrected visual acuity in the the left eye, ELM: external limiting membrane, EZ: inner segment ellipsoid zone, CIZ: cone interdigitation zone

A 38-year-old man was referred to our hospital because of recurrent panuveitis in both eyes. Recurrent oral ulcer, folliculitis, and genital ulcer were noted as systemic complications. He had been treated with topical and systemic corticosteroids as needed for 5 years prior to presentation. On ophthalmologic examination, best corrective visual acuity (BCVA) was 20/60 in the right and 20/200 in the left eye, and intraocular pressures were 12 and 11 mmHg, respectively. Moderate cell infiltration into the anterior chamber (+2 cells), and diffuse vitritis (+1-2 cells) were observed in both eyes, but no definite retinal lesions were detected (Fig. 1a and b). However, fluorescein angiography (FA) showed severe dye leakage from extensive retinal vessels at the optic disc, macula (Fig. 1c and d) and peripheral retina (Fig. 1e and f) in both eyes. Spectral domain-optical coherence tomography (SD-OCT) revealed retinal cysts and disruption of ELM, EZ, and CIZ in the macular region of both eyes (Fig. 2a). BD was diagnosed based on the ocular features and systemic lesions. Since visual acuity in both eyes was being deteriorated by persistent ocular inflammation despite corticosteroid treatment, infliximab therapy was initiated. After 3 months of infliximab therapy, although foveal excavation was first recovered with disappearance of macular edema in both eyes, the disrupted outer retinal layers did not improve and BCVA remained unchanged (Fig. 2b). However, after 12 months of infliximab therapy, ELM and EZ were well defined in both eyes (Fig. 2c) and BCVA improved to 20/40 in the right eye and 20/30 in the left eye. Finally, the CIZ became distinguishable after 24 months of infliximab therapy, and BCVA in both eyes was 20/25. Vasculitis indicated by dye leakage on FA remained only in peripheral retina, and ocular inflammatory attack did not recur in both eyes after the initiation of infliximab therapy.

## Discussion

Disappearance of ELM and EZ in BD-associated uveitis has been already reported [2]. Severe dye leakage from extensive retinal vessels on FA is characteristic of BD-associated uveitis, however dye leakage from choroidal vessels, choroidal filling defect, and irregular filling of the choriocapillaries are also observed by indocyanine green angiography [3]. Since choroidal circulation providing alimentation and oxygen to sensory retina, these choroidal lesions would induce degeneration of the photoreceptor layer shown by disappearance of ELM and EZ. Although it is known that disruption of ELM, EZ and CIZ in epiretinal membrane (ERM), macular hole (MH), and fovea-off retinal detachment (RD) are reconstituted after surgery [4–6], it has not been verified in BD-associated uveitis. The present case illustrates that disrupted ELM, EZ, and CIZ at the fovea caused by BD-associated uveitis can be restored through stable resolution of ocular inflammation by infliximab therapy, although it is unclear whether the reconstitution is a result of regeneration or recovery of photoreceptor cells.

The correlation between the integrity of ELM, EZ and CIZ bands and visual acuity was investigated by many studies. In the present case, full recovery of BCVA was accompanied by the appearance of CIZ, which lagged behind recovery of the integrity of ELM and EZ bands. In ERM and MH, restoration of CIZ correlates the most strongly with postoperative BCVA [5, 6]. On the other hand, the integrity of ELM is considered a prerequisite in predicting subsequent EZ and CIZ restoration and VA recovery, and the lack of the ELM line is suggested to reflect irreversible damage or loss of photoreceptor cells after RD [4]. In the present case, although ELM was not visualized before and after 3 month of infliximab therapy in the left eye, continuous ELM line appeared at 6 months earlier than reconstitution of EZ or CIZ.

## Conclusions

In conclusion, we have found in the present case that continuous remission of BD-associated uveitis by infliximab treatment possibly restores morphological changes in the retina which are revealed by disappearance or disruption of ELM, EZ, and CIZ on SD-OCT, which led to the improvement of visual acuity.

## Consent

Written informed consent was obtained from the patient for publication of this Case Report and accompanying images. A copy of the written consent is available for review by the Editor of this journal.

### Availability of supporting data

The fundus photos and serial optical coherence tomography images supporting the results of this article are included within the article nd its additional files named Figs. 1 and 2.

## Abbreviations

BD: Behçet’s disease
SD-OCT: Spectral domain-optical coherence tomography
ELM: External limiting membrane
EZ: Inner segment ellipsoid zone
CIZ: Cone interdigitation zone
